# DNA G-Wire Formation Using an Artificial Peptide is Controlled by Protease Activity

**DOI:** 10.3390/molecules22111991

**Published:** 2017-11-16

**Authors:** Kenji Usui, Arisa Okada, Shungo Sakashita, Masayuki Shimooka, Takaaki Tsuruoka, Shu-ichi Nakano, Daisuke Miyoshi, Tsukasa Mashima, Masato Katahira, Yoshio Hamada

**Affiliations:** 1 Faculty of Frontiers of Innovative Research in Science and Technology (FIRST), Konan University, 7-1-20 Minatojima-minamimachi, Chuo-ku, Kobe 650-0047, Japan; a.okada910311@gmail.com (A.O.); s1491013@s.konan-u.ac.jp (S.S.); ma_kunn7536548426@yahoo.co.jp (M.S.); tsuruoka@center.konan-u.ac.jp (T.T.); shuichi@center.konan-u.ac.jp (S.N.); miyoshi@center.konan-u.ac.jp (D.M.); pynden@gmail.com (Y.H.); 2 Institute of Advanced Energy, Kyoto University, Gokasho, Uji, Kyoto 611-0011, Japan; mashima0@iae.kyoto-u.ac.jp (T.M.) katahira@iae.kyoto-u.ac.jp (M.K.); 3 Graduate School of Energy Science, Kyoto University, Gokasho, Uji, Kyoto 611-0011, Japan

**Keywords:** designed peptide, G-wire, G-quadruplex, protease, peptide nucleic acid, PNA

## Abstract

The development of a switching system for guanine nanowire (G-wire) formation by external signals is important for nanobiotechnological applications. Here, we demonstrate a DNA nanostructural switch (G-wire <--> particles) using a designed peptide and a protease. The peptide consists of a PNA sequence for inducing DNA to form DNA–PNA hybrid G-quadruplex structures, and a protease substrate sequence acting as a switching module that is dependent on the activity of a particular protease. Micro-scale analyses via TEM and AFM showed that G-rich DNA alone forms G-wires in the presence of Ca^2+^, and that the peptide disrupted this formation, resulting in the formation of particles. The addition of the protease and digestion of the peptide regenerated the G-wires. Macro-scale analyses by DLS, zeta potential, CD, and gel filtration were in agreement with the microscopic observations. These results imply that the secondary structure change (DNA G-quadruplex <--> DNA/PNA hybrid structure) induces a change in the well-formed nanostructure (G-wire <--> particles). Our findings demonstrate a control system for forming DNA G-wire structures dependent on protease activity using designed peptides. Such systems hold promise for regulating the formation of nanowire for various applications, including electronic circuits for use in nanobiotechnologies.

## 1. Introduction

Switchable and controllable molecular devices have the potential to play key roles in nanomaterials and nanomachines, whose structure and function can be regulated in a dynamic manner [1,2,3,4,5,6,7,8]. DNA molecules are attractive for the design of such nanodevices because they have periodic one-, two-, and three-dimensional nanostructures, and they can be easily designed logically and systematically based on complementary binding [9,10,11,12,13,14,15,16,17,18,19,20]. Guanine nanowires (G-wires) [21,22,23,24] in particular are attracting interest as functional elements in molecular electronics and nanotechnology because of their unique optical and electrochemical characteristics [25,26,27]. G-wire is an alternative functional DNA nanostructure based on a four-stranded DNA helix called a G-quadruplex [28,29,30]. The G-quadruplex is formed by guanine-rich DNA sequences such as telomere DNAs with cyclic Hoogsteen base pairs between four guanine bases in a coplanar arrangement [31,32,33,34]. This structure regulates cellular events [35,36,37,38,39,40,41], such as transcription and telomerase elongation, which play roles in various serious diseases and cellular aging. G-wires have recently been used to generate nanocircuits, DNA computers, and nanotechnological materials [42,43,44]. Therefore, the regulation of the polymorphic nature of the G-quadruplex and switching G-wire formation present a novel methodology for both developing molecular devices in vitro and for controlling biological phenomena in vivo.

**Figure 1:**
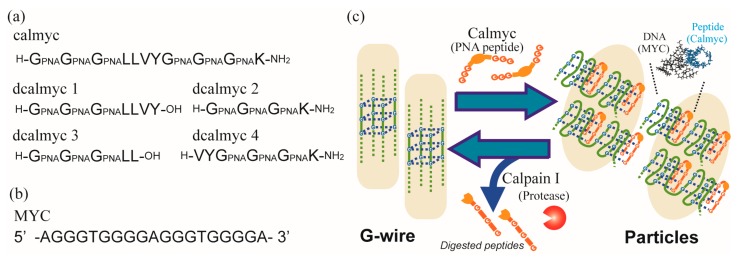
(**a**) Sequences of the designed peptides used in this study; (**b**) Sequence of the DNA used in this study; (**c**) Scheme showing switching of DNA nanostructures with changes of DNA secondary structures by the designed peptide, calmyc, and a specific protease, calpain I.

We previously constructed a regulation system for the DNA secondary structure formation of G-rich sequences, using a designed peptide nucleic acid (PNA) [45,46,47,48,49,50,51], whose functionality could be switched through protease activity [52] (Figure 1 and Figure S1). Furthermore, this study implied that it was theoretically possible that G-wire structures constructed using this peptide could be changed to other nanostructures, and that the G-wire structure could be regenerated by the protease (Figure 1c). In the present study, we use micro-scale imaging techniques, transmission electron microscopy (TEM) and atomic force microscopy (AFM), and macro-scale analytical techniques, zeta potential, dynamic light scattering (DLS), circular dichroism (CD), nuclear magnetic resonance (NMR), and gel filtration, to demonstrate the structural switching of a DNA nanostructure. This nanostructure is constructed using a designed PNA peptide that exhibits a switching functionality that is dependent on protease activity. Our findings suggest that a secondary structure change in the G-wires (higher-order structure) using an artificial peptide induces the conformational change into a different nanostructure (higher-order structure).

## 2. Results and Discussion

The PNA–peptide (calmyc) is composed of two parts (Figure 1a and Figure S1a). One part consists of guanine PNA-rich sequences that form hetero-quadruplexes with homologous DNA oligomers (Figure 1b and Figure S1b). The on-to-off switching module is regulated by the activity of a particular protease (calpain I) and mimics the protease substrate sequence. We previously demonstrated that the designed peptide could bind to DNA and form a hetero-quadruplex structure in the absence of the protease, whereas in the presence of the protease, the conjugates were digested to provide the peptides dcalmyc 1–4 (Figure 1a and Figure S1a). Concurrently, the hetero-quadruplexes lost their binding ability, resulting in the collapse of the DNA–PNA hetero-quadruplex structure (Figure S1c). We previously used a G-rich DNA sequence from the promoter region of human proto-oncogenes (MYC from c-MYC [53,54], Figure 1b and Figure S1b) and showed that calmyc binds to MYC and forms a hetero-quadruplex structure, and that the conjugate is digested and simultaneously loses its binding ability upon the addition of calpain I.

**Figure 2:**
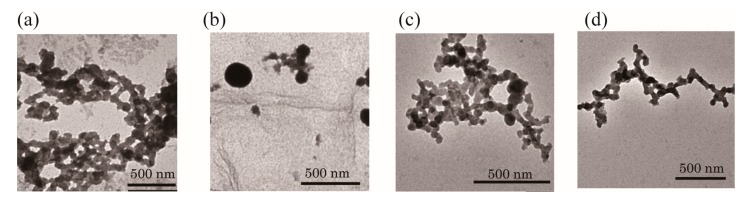
TEM images of the samples after incubation with Ca^2+^ for 3 h with (**a**) MYC alone; (**b**) MYC and calmyc; (**c**) MYC, calmyc, and calpain I, and after incubation with Ca^2+^ for 24 h with MYC, calmyc, and calpain I (**d**). The TEM samples were stained with phosphotungstic acid.

In the present study, we determined whether the MYC G-rich DNA sequence could form a G-wire in the presence of Ca^2+^ checking by TEM (Figure 2a and Figure S2a,b). Incubation of MYC in the presence of Ca^2+^ for 3 h resulted in the generation of G-wires observable by TEM (Figure 2a and Figure S2b). To determine whether MYC could switch from a G-wire to another nanostructure by the addition of calmyc, MYC, and calmyc were incubated in the presence of Ca^2+^ for 3 h. Figure 2b and Figure S2d show the absence of wire structures, and particles of random shapes and sizes can be found. Calmyc alone provided smaller-sized particles, as shown in Figure S2c. These results indicated that calmyc could prevent MYC from forming G-wire structures and could form particles with MYC. The individual addition of dcalmyc 1 and 2, or dcalmyc 3 and 4, which are calmyc fragments produced by the digestion of calmyc with calpain I, to MYC provided structures similar to that of MYC alone (Figure S2e,f). This result indicates that the digestion of calmyc abolished the ability to bind to MYC, resulting in the collapse of the DNA–PNA hetero-quadruplex structure and the formation of MYC G-wire structures, as expected. Further, we demonstrated G-wire formation in a mixture of MYC and calmyc upon the addition of calpain I. After 3 h or 24 h enzyme reaction and incubation, MYC provided structures similar to those of MYC alone or MYC with dcalmyc 1–4, as expected (Figure 2c,d and Figure S2g,h). This also indicated that digestion of calmyc abolished its ability to bind to MYC, resulting in the loss of ability to form the DNA–PNA hetero-quadruplex structure.

**Figure 3:**
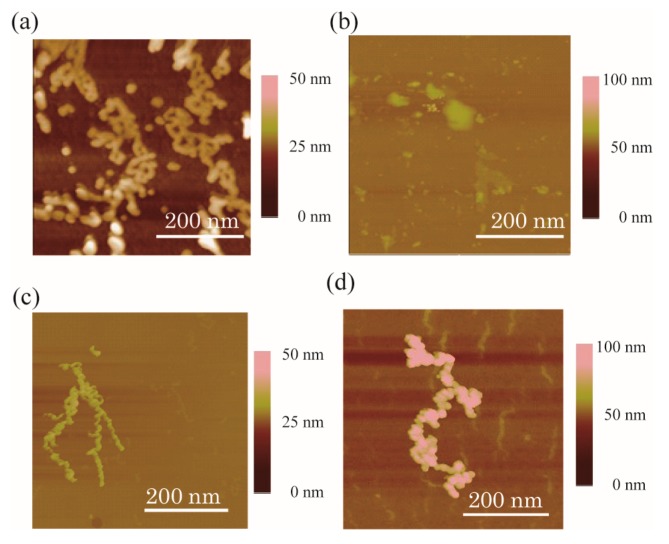
AFM images of the samples after incubation with Ca^2+^ for 3 h with (**a**) MYC alone; (**b**) MYC and calmyc; and (**c**) MYC, calmyc, and calpain I and (**d**) after incubation with Ca^2+^ for 24 h with MYC, calmyc, and calpain I.

We additionally conducted AFM analyses and compared the results to those obtained with TEM (Figure 3 and Figure S3). Following staining, MYC incubated with Ca^2+^ for 3 h without calmyc showed wire-shaped deposits, whereas MYC plus calmyc and Ca^2+^ produced randomly sized particles. The addition of 1 dcalmyc and 2, or dcalmyc 3 and 4 (calmyc digested peptides with calpain I), to MYC provided G-wire structures similar to that of MYC alone. The addition of calpain I to a mixture of MYC and calmyc in the presence of Ca^2+^ for 3 h or 24 h resulted in the generation of G-wire structures. All of these results agreed with the TEM results.

These observations were verified by five macro-observation techniques, electrophoresis, DLS, zeta potential, CD, NMR, and gel filtration. The binding of the calmyc peptide and MYC had been assayed using electrophoresis in our previous paper [52]. Briefly, we checked that calmyc could bind to MYC, resulting in the cationic PNA peptide causing the bands to migrate with a higher range of apparent molecular weights than expected.

**Figure 4:**
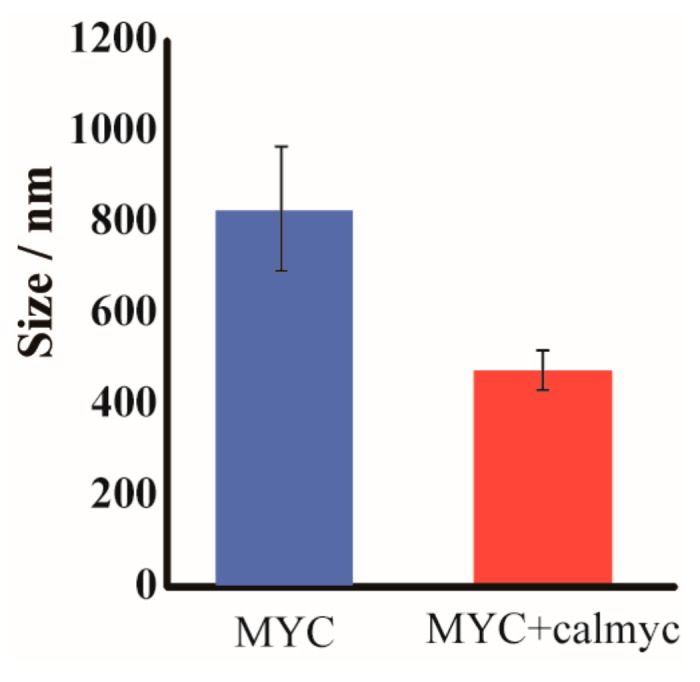
DLS analysis of the size of MYC incubated with Ca^2+^ for 3 h with or without calmyc.

Then, we conducted DLS analysis and compared the results to those obtained with TEM and AFM. MYC incubated with Ca^2+^ for 3 h with or without calmyc-produced structures of expected sizes upon DLS analysis (i.e., MYC incubated with Ca^2+^ for 3 h without calmyc-produced complexes of around 900 nm and MYC incubated with Ca^2+^ for 3 h with calmyc-produced complexes of around 500 nm; Figure 4). These macroscopic analyses were in agreement with microscopic observations (TEM and AFM results) (Figure 2 and Figure 3, Figures S2 and S3).

**Figure 5:**
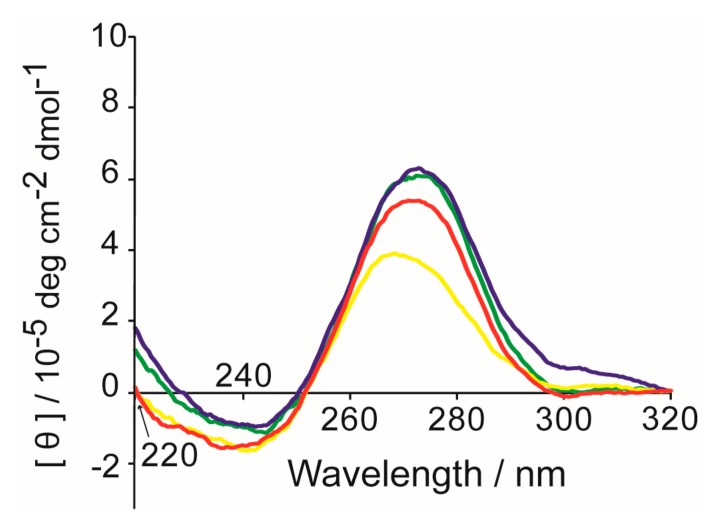
CD spectra of 1 μM MYC alone (red), 1 μM MYC and 2 μM calmyc (yellow), and 1 μM MYC and 2 μM dcalmycs (blue: dcalmyc 1 and 2; green: dcalmyc 3 and 4) in a buffer containing 100 mM CaCl2, 100 mM NaCl, and 20 mM Tris-HCl (pH 7.0) after 3 h of incubation at 45 °C.

**Figure 6:**
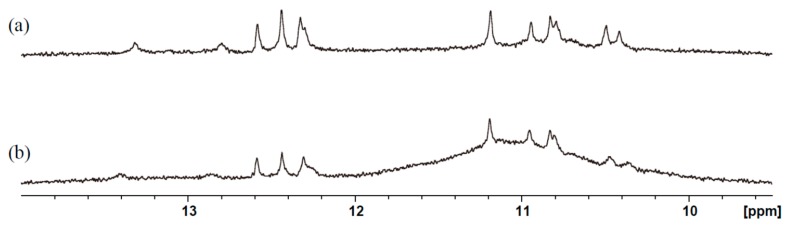
Imino proton regions of ^1^H-NMR spectra of 50 µM MYC alone (**a**) or with 100 µM calmyc (**b**).

MYC alone, a mixture of MYC and calmyc, and a mixture of dcalmyc 1–4 and MYC, were incubated in the presence of Ca^2+^ for 3 h, after which far-UV CD spectroscopy was carried out on the samples (Figure 5). The spectrum of MYC alone was similar to that of other parallel G-quadruplexes previously described, including a positive maximum ellipticity at 260 nm [22,55,56,57]. MYC and calmyc incubated together resulted in a small change in the position of the positive maximum peak (a positive maximum ellipticity at 257 nm). The presence of dcalmycs did not cause significant changes in the position of the positive maximum peak. The CD results indicate that the secondary structure of the calmyc-MYC hybrid in the presence of Ca^2+^ slightly differs from that of MYC alone, but this small difference causes significant changes in the nanostructure. An NMR sample of MYC with Ca^2+^ exhibited precipitation. It is supposed that the precipitation of the G-wire was formed due to high MYC concentration applied for the NMR sample. Thus, further analysis of the G-wire by NMR was not feasible. In the absence of Ca^2+^, MYC gave relatively sharp NMR signals in the imino proton region (Figure 6). The signals characteristic to a G-quadruplex were observed at 10.2–11.2 ppm [58,59,60,61]. Additionally, other signals were also observed at 12.2–13.2 ppm. These results suggest that MYC formed a certain structure with the G-quadruplex and several non-Watson–Crick base pairs in the absence of Ca^2+^. When calmyc was added, a broad signal appeared around at 10–12 ppm (Figure 6b). The broad signal may originate from the intermediate exchange in an NMR time scale between multiple different kinds of complexes comprising MYC and calmyc. Alternatively, the broad signal may originate from a relatively large complex comprising MYC and calmyc. In any case, the chemical shift value of 10–12 ppm for the broad peak implies that the formed complex include the G-quadruplex. Thus, it is suggested that the addition of calmyc resulted in the formation of the complex that includes the G-quadruplex. The relatively sharp peaks of MYC are also present in the presence of calmyc (Figure 6b). This suggests that some fraction of MYC takes an original certain structure.

**Figure 7:**
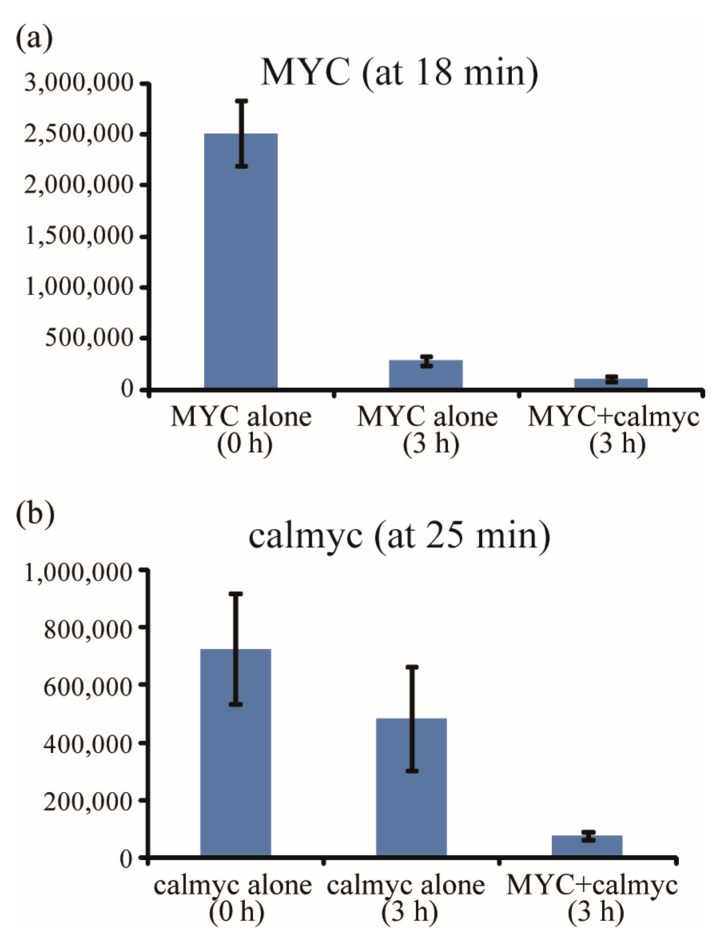
Peak areas obtained from gel filtration analysis. Monomer peak areas of (**a**) MYC (at 13.5 mL) and (**b**) calmyc (at 18.75 mL) following 0 h of incubation of MYC alone (MYC alone (0 h)) or calmyc alone (calmyc alone (0 h)), 3 h of incubation of MYC alone (MYC alone (3 h)) or calmyc alone (calmyc alone (3 h)), and 3 h of incubation of MYC and calmyc together (MYC + calmyc (3 h)).

**Figure 8:**
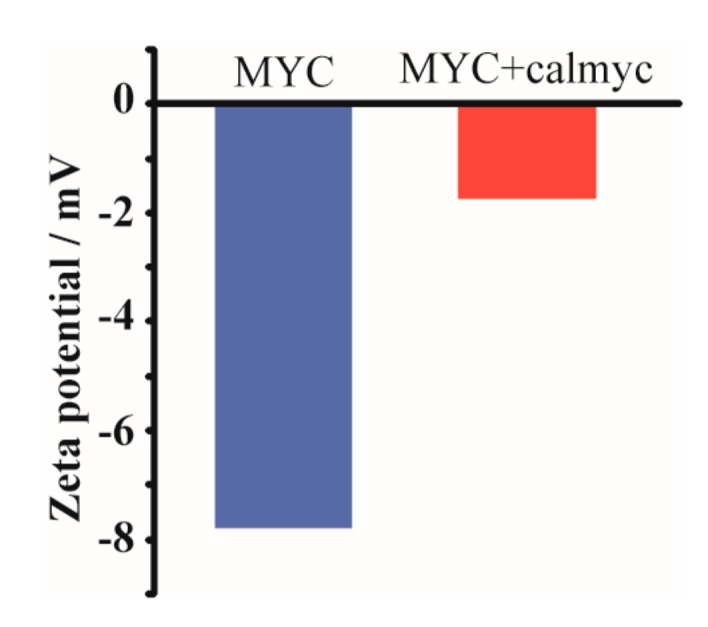
Zeta potentials of MYC incubated with Ca^2+^ for 3 h with or without calmyc.

The gel filtration results in Figure 7 showed monomer peak areas (the amounts of unstructured DNA or peptide in a sample) for MYC (a peak area at 13.5 mL, Figure 7a) and calmyc (a peak area at 18.75 mL, Figure 7b) when the samples with MYC alone (MYC), calmyc alone (calmyc), or MYC plus calmyc (MYC + calmyc) were incubated for 0 or 3 h with Ca^2+^. A 3 h incubation of MYC or calmyc sample in the presence of Ca^2+^ (MYC alone (3 h) and calmyc alone (3 h)) showed dramatically smaller areas than both areas of monomer peaks at 0 h (MYC alone (0 h) and calmyc alone (0 h)). In contrast, a 3 h incubation of MYC and calmyc with Ca^2+^ (the sample of MYC + calmyc (3 h)), compared to a 3 h incubation of MYC or calmyc alone with Ca^2+^ (MYC alone (3 h) or calmyc alone (3 h)), resulted in areas in both monomer peaks of MYC (Figure 7a) and calmyc (Figure 7b) that were even smaller. Zeta potential measurements further confirmed calmyc bound to MYC and these formed particles. As shown in Figure 8, a negative zeta potential value (−7.78 mV) could be seen in the G-wire structure of MYC incubated with Ca^2+^ for 3 h, as expected. Whereas a smaller negative value of −1.74 mV was shown in MYC incubated with Ca^2+^ for 3 h with calmyc, indicating that the nanoparticles were composed of negative-charged MYC and positive-charged calmyc (Figure 1). These results implied that calmyc bound to MYC and that this complex then assembled and formed a nanoparticle, which agrees with the micro-scale technique results, AFM and TEM.

**Figure 9:**
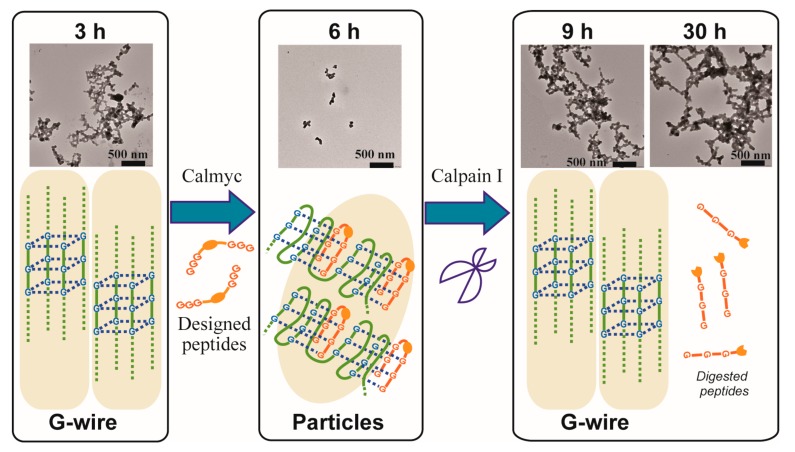
TEM images of reversible switching of DNA nanowire formation with time using the peptide and the protease. MYC alone in the presence of Ca^2+^ was incubated for 3 h (the 3 h TEM image in the left column), the peptide was then added, and the sample was incubated for another 3 h (the 6 h TEM image in the middle column). After a total of 6 h of incubation, calpain I was added, and the sample was incubated for another 3 h or 24 h (the 9 h TEM image and the 30 h TEM image in the right column).

Finally, the stepwise switching of DNA nanowires using MYC and calpain I was observed via TEM (Figure 9) and AFM (Figure S4). MYC formed G-wires in the presence of Ca^2+^ after 3 h of incubation without calmyc. Subsequent addition of calmyc and further incubation for 3 h resulted in the G-wires being broken, in agreement with the one-step results observed via TEM and AFM (Figure 2b and Figure 3b). Additionally, in this condition, particles with ca. 20–50 nm in diameter could be seen, indicating that we could control the particle diameters by changing conditions such as the incubation time and concentration. Finally, calpain I was added to the sample and the sample was incubated for 3 or 24 additional hours. This resulted in the digestion of calmyc and regeneration of the G-wire structure, again in agreement with the one-step results observed via TEM and AFM (Figure 2c,d and Figure 3c,d). We therefore demonstrated reversible changes in DNA nanowire formation using a PNA-peptide and a protease.

## 3. Materials and Methods 

### 3.1. General Remarks

All chemicals and solvents were of reagent or HPLC grade and were used without further purification. Oligodeoxynucleotide samples purified by HPLC were purchased from Hokkaido System Science (Sapporo, Japan). HPLC was performed on a GL-7400 HPLC system (GL Sciences, Tokyo, Japan) using an Inertsil ODS-3 (10 × 250 mm; GL Science, Tokyo, Japan) column for preparative purification with a linear acetonitrile/0.1% trifluoroacetic acid (TFA) gradient at a flow rate of 3.0 mL/min. Peptides were analyzed using MALDI-TOF MS on an Autoflex III (Bruker Daltonics, Billerica, MA, USA) mass spectrometer with 3,5-dimethoxy-4-hydroxycinnamic acid as the matrix. Amino acid analysis was carried out using an Inertsil ODS-2 (4.6 × 200 mm; GL Science, Tokyo, Japan) after hydrolysis in 6 M HCl at 110 °C for 24 h in a sealed tube, followed by phenyl isothiocyanate labeling.

### 3.2. Synthesis of Artificial Peptides

The designed peptides except for dcalmyc 1 and 3 were synthesized manually on Fmoc-NH-SAL-PEG resin (Watanabe Chemical Industries, Hiroshima, Japan) by Fmoc chemistry [62] using Fmoc-AA-OH (4 eq., Watanabe Chemical Industries, Hiroshima, Japan) and Fmoc PNA monomers (4 eq., Panagene, Daejeon, Korea) according to the O-(7-azabenzotriazol-1-yl)-1,1,3,3-tetramethyluronium hexafluorophosphate (HATU, Watanabe Chemical Industries, Hiroshima, Japan) method. dcalmyc 1 and 3 were synthesized by Fmoc chemistry on Wang resin (Watanabe Chemical Industries, Hiroshima, Japan) to which the first coupling amino acid, Fmoc-Tyr(tBu)-OH or Fmoc-Leu-OH respectively, was attached using the 1,3-diisopropylcarbodiimide (DIC) and *N*,*N*-dimethyl-aminopyridine (DMAP) method. The side chain protecting groups were as follows: *t*-butyl (*t*Bu) for Tyr, *t*-butyloxycarbonyl (Boc) for Lys, and benzhydryloxycarbonyl (Bhoc) for guanine PNA monomers. The peptides were cleaved from the resins and the side chain protecting groups were removed by incubating the peptide-resins for 2 h in TFA (Watanabe Chemical Industries, Hiroshima, Japan)/H_2_O/triisopropylsilane (Wako Pure Chemical Industries, Tokyo, Japan) (20:1:1, *v*/*v*/*v*). The peptides were precipitated by the addition of cold diethyl ether, collected by centrifugation, purified by RP-HPLC, and characterized by amino acid analysis and MALDI-TOF MS: calmyc, *m*/*z* 2380.0 ([M + H]^+^ calcd. 2381.4); dcalmyc1, *m*/*z* 1382.5 ([M + H]^+^ calcd. 1381.5); dcalmyc2, *m*/*z* 1019.0 ([M + H]^+^ calcd. 1019.0); dcalmyc 3, *m*/*z* 1118.1 ([M + H]^+^ calcd. 1119.1); dcalmyc 4, *m*/*z* 1281.1 ([M + H]^+^ calcd. 1281.3). Purified peptides were dissolved in MilliQ water to about 300 μM. Their concentrations were measured via amino acid analysis, and they were then stored at 4 °C.

### 3.3. Atomic Force Microscope Measurements

Atomic force microscope (AFM) measurements were carried out after incubating 25 μM MYC with/without 50 μM calmyc or 20 units calpain I at 45 °C in a buffer containing 100 mM NaCl, 20 mM Tris (pH 7.0) with or without 100 mM CaCl_2_. A 20 μL sample was deposited onto freshly cleaved mica washed three times with 20 μL MilliQ water, and then dried with a stream of N_2_ gas. AFM images were obtained in the tapping mode using a Nanoscope III (Digital Instruments Inc., Santa Barbara, CA, USA).

### 3.4. Transmission Electron Microscope Measurements 

After incubation, a 20 μL sample was placed on a TEM grid (Cu 200 mesh covered with a Nisshin EM collodion membrane, Nisshin, Japan) for 1 min and dried with a filter paper. MilliQ water (20 µL) was then placed on the grid for 1 min and absorbed with filter paper. This process was repeated three times to remove salts from the sample. Then, 2% phosphotungstic acid solution was placed on the grid for 1 min and the MilliQ washing process was repeated three times. All samples were dried in vacuo before TEM measurements. The samples were characterized by TEM operated at 120 kV using a JEOL JEM-1400 electron microscope.

### 3.5. Dynamic Light Scattering (*DLS*) Measurements and Zeta Potential Measurements

Sample solution (40 μL) was transferred into a UV-transparent disposable cuvette S3 (Sarstedt, Tokyo, Japan) for DLS measurements, and sample solution (800 μL for zeta potential) was transferred into a folded capillary cell DTS1070 (Malvern Instruments, Worcestershire, UK) for zeta potential measurements. DLS and zeta potential data were acquired on a Zetasizer ZEN3600 instrument (Sysmex, Kobe, Japan) equipped with a 633 nm laser.

### 3.6. Circular Dichroism (CD) Spectroscopy

CD spectroscopy was performed at room temperature using DNA (1 μM) and the peptides (0 or 2 μM) in 100 mM CaCl_2_, 100 mM NaCl, and 20 mM Tris-HCl (pH 7.0). A J-820 spectropolarimeter (JASCO, Tokyo, Japan) with a thermoregulator and a quartz cell with a 1 cm pathlength was used.

### 3.7. NMR Spectroscopy

NMR samples were dissolved in 10 mM Tris-HCl (pH 7.5) containing 0.1 mM 4,4-dimethyl-4-silapentane-1-sulfonic acid (DSS). NMR spectra were recorded at 25 °C with Bruker DRX 600 and AVANCE III HD 600 spectrometers equipped with a cryoprobe with a Z-gradient. 1H chemical shift was calibrated with a resonance of DSS. Spectra were processed and analyzed with XWIN-NMR/ TopSpin (Bruker, Billerica, MA, USA).

### 3.8. Size-Exclusion Chromatography (SEC) Measurements

Before SEC measurements, samples comprising 25 μM MYC with/without 50 μM calmyc were incubated at 45 °C for 0 or 3 h in a buffer containing 100 mM CaCl_2_, 100 mM NaCl, and 20 mM Tris-HCl (pH 7.0). SEC was performed using an HPLC system equipped with a SuperdexTM 75 10/300 column (bed volume: ca. 24 mL) (GE Healthcare, Tokyo, Japan) at 25 °C. The flow rate was 0.75 mL/min, the samples were eluted using the same buffer and elution was monitored by measuring the absorbance at 260 nm.

## 4. Conclusions

We have developed a switching system for G-wire formation through external signals using a designed PNA–peptide conjugate. The micro-scale techniques, TEM and AFM, and the macro-scale techniques, DLS, zeta potential, CD, NMR, and gel filtration, were used to show the reversible changes in DNA nanowire formation using a PNA–peptide and a protease. This study also suggests that changes in secondary structure induce changes in a well-formed nanostructure (higher-order structural changes). This approach is not limited to switching by protease activity, described in this study. Substitution of the protease substrate sequence with other enzymatic substrate sequences or artificial moieties such as photo-switching and chemo-switching functional groups could further provide a variety of well-controlled switching nanodevices [63,64,65,66]. Such systems hold promise for regulating the formation of nanowire structures for various applications, including electronic circuits for use in nanotechnologies and nanobiotechnologies.

## Supplementary Materials

The following are available online. Sequences of the peptides and scheme of this study, TEM and AFM images of MYC and the peptides.
