# Supplementary material for: DNA G-Wire Formation Using an Artificial Peptide is Controlled by Protease Activity
Source: Molecules. 2017 Nov 16;22(11):1991. doi: 10.3390/molecules22111991 (PMC6150327; doi:10.3390/molecules22111991)
Supplement: Supplementary file 1 [file molecules-22-01991-s001.pdf]

molecules

DNA G-Wire Formation Using an Artificial Peptide is Controlled by Protease Activity

Kenji Usui [1,*], Arisa Okada [1], Shungo Sakashita [1], Masayuki Shimooka [1], Takaaki Tsuruoka [1], Shu-ichi Nakano [1], Daisuke Miyoshi [1], Tsukasa Mashima [2,3], Masato Katahira [2,3] and Yoshio Hamada [1]

[1] Faculty of Frontiers of Innovative Research in Science and Technology (FIRST), Konan University, 7-1-20 Minatojima-minamimachi, Chuo-ku, Kobe 650-0047, Japan; a.okada910311@gmail.com (A.O.); s1491013@s.konan-u.ac.jp (S.S.); ma_kunn7536548426@yahoo.co.jp (M.S.); tsuruoka@center.konan-u.ac.jp (T.T.); shuichi@center.konan-u.ac.jp (S.N.); miyoshi@center.konan-u.ac.jp (D.M.); pynden@gmail.com (Y.H.)

[2] Institute of Advanced Energy, Kyoto University, Gokasho, Uji, Kyoto 611-0011, Japan; mashima0@iae.kyoto-u.ac.jp (T.M.) katahira@iae.kyoto-u.ac.jp (M.K.)

[3] Graduate School of Energy Science, Kyoto University, Gokasho, Uji, Kyoto 611-0011, Japan

* Correspondence: kusui@center.konan-u.ac.jp; Tel.: +81-78-303-1418

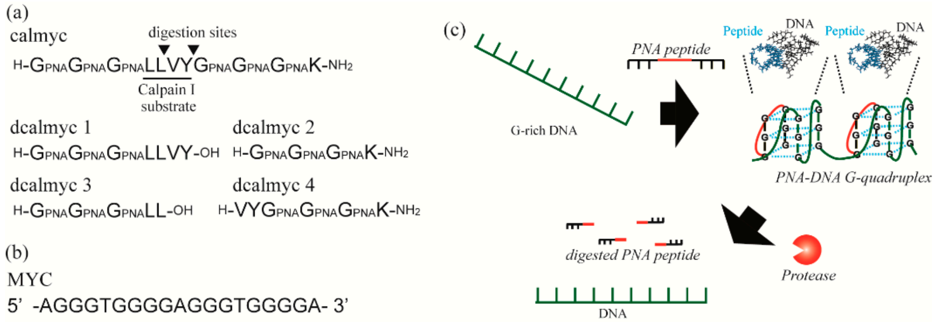

Figure S1. (**a**) Sequences of the peptides used in this study. (**b**) Sequence of the DNA used in this study. (**c**) Scheme showing switching of DNA secondary structure by the designed peptide and a specific protease. This figure was modified and reproduced from Ref. 52 with permission from the Royal society of chemistry.

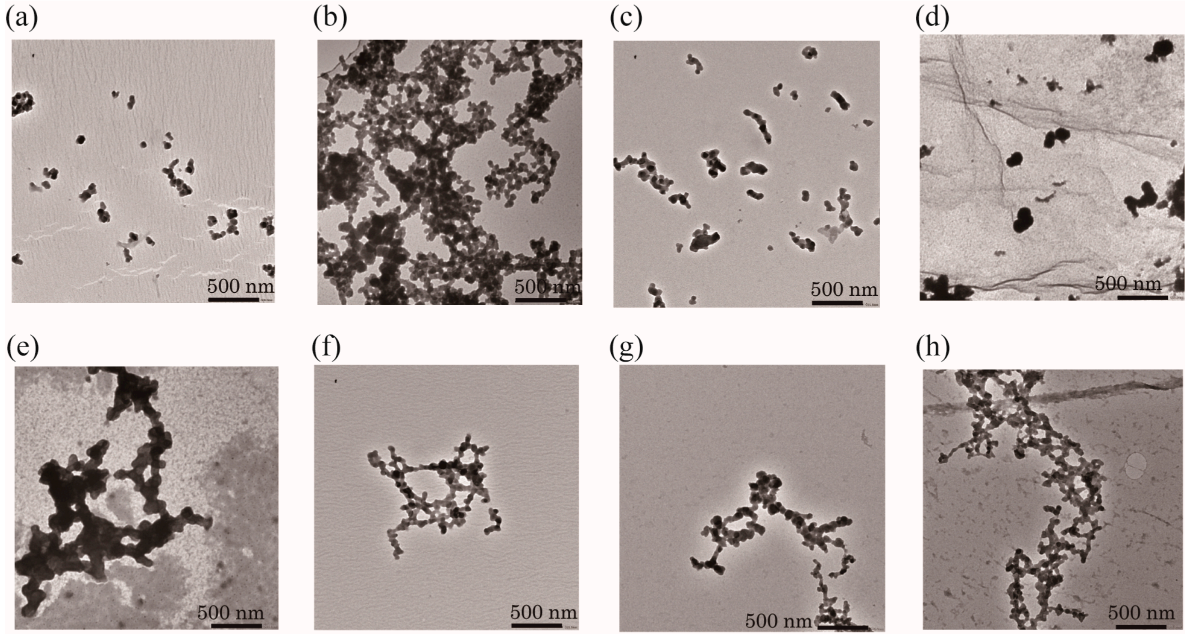

Figure S2. (**a**) TEM image following incubation for 3 hours with MYC in the absence of Ca^{2+}. TEM images following incubation for 3 hours in the presence of Ca^{2+} with (**b**) MYC alone (an alternative

image of Figure 2a), (**c**) calmyc alone, (**d**) MYC and calmyc (an alternative image of Figure 2b), (**e**) MYC and dcalmyc 1,2, (**f**) MYC and dcalmyc 3,4, (**g**) MYC, calmyc, and calpain I (an alternative image of Figure 2c), (**h**) following incubation for 24 hours in the presence of Ca^{2+} with MYC, calmyc, and calpain I (an alternative image of Figure 2d).

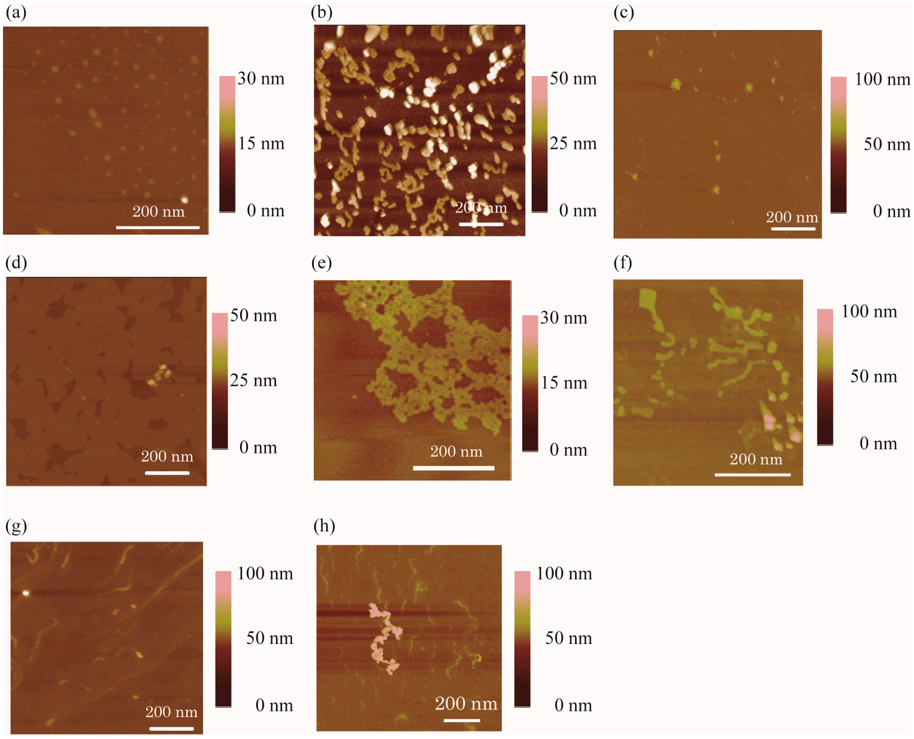

Figure S3. (a) AFM image following incubation for 3 hours with MYC in the absence of Ca^{2+}. AFM images following incubation for 3 hours in the presence of Ca^{2+} with **(b)** MYC alone (an alternative image (wider range) of Figure 2a), **(c)** calmyc alone, **(d)** MYC and calmyc (an alternative image (wider range) of Figure 2b), **(e)** MYC and dcalmyc 1,2, **(f)** MYC and dcalmyc 3,4, **(g)** MYC, calmyc, and calpain I (an alternative image (wider range) of Figure 2c), **(h)** following incubation for 24 hours in the presence of Ca^{2+} with MYC, calmyc, and calpain I (a wider range image of Figure 2d).

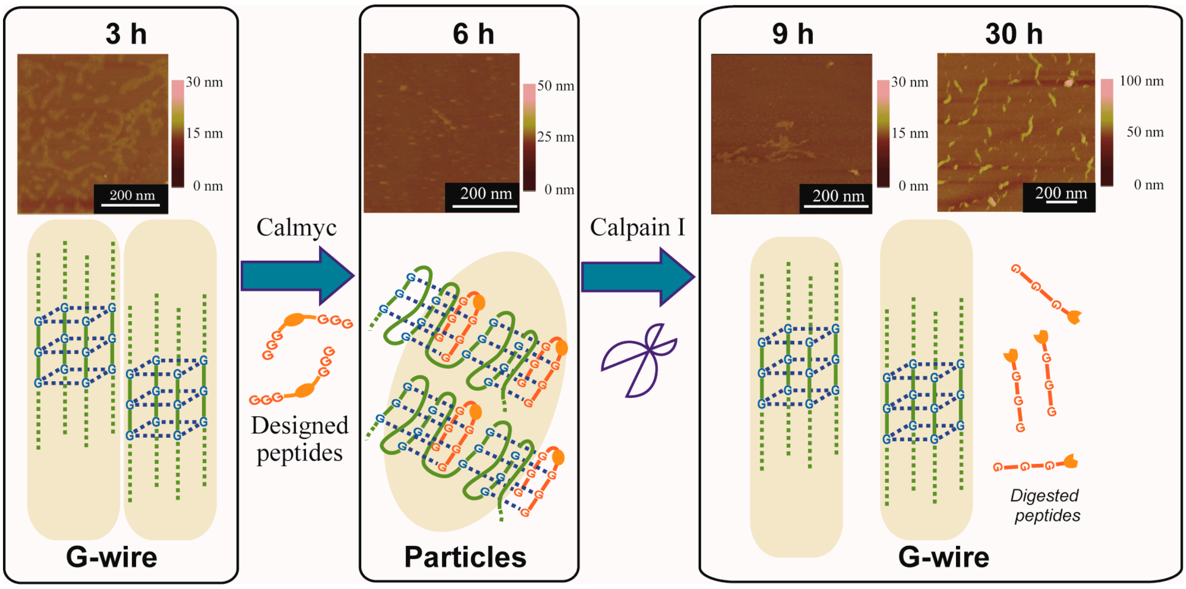

Figure S4. AFM images of reversible switching of DNA nanowire formation with time using the peptide and the protease. MYC alone in the presence of Ca^{2+} was incubated for 3 hours (the 3 h AFM image in the left column), then the peptide was added and the sample was incubated for another 3

hours (the 6 h AFM image in the middle column). After a total of 6 hours of incubation, calpain I was added and the sample was incubated for another 3 hours or 24 hours (the 9 h AFM image and the 30 h AFM image in the right column).
